# Static Magnetic Fields Reduce Oxidative Stress to Improve Wound Healing and Alleviate Diabetic Complications

**DOI:** 10.3390/cells11030443

**Published:** 2022-01-27

**Authors:** Chuanlin Feng, Biao Yu, Chao Song, Junjun Wang, Lei Zhang, Xinmiao Ji, Ying Wang, Yanwen Fang, Zhongcai Liao, Min Wei, Xin Zhang

**Affiliations:** 1 Institutes of Physical Science and Information Technology, Anhui University, Hefei 230039, China; fcl@mail.ustc.edu.cn; 2 High Magnetic Field Laboratory, Hefei Institutes of Physical Science, Chinese Academy of Sciences, Hefei 230031, China; biaoyu@hmfl.ac.cn (B.Y.); chaosong@hmfl.ac.cn (C.S.); junjunwang1222@hmfl.ac.cn (J.W.); leizhang@hmfl.ac.cn (L.Z.); xinmiaoji@hmfl.ac.cn (X.J.); wy1999hf@mail.ustc.edu.cn (Y.W.); 3 Science Island Branch of Graduate School, University of Science and Technology of China, Hefei 230026, China; 4 Heye Health Technology Co., Ltd., Huzhou 313300, China; yanwen.fang@heaye.com (Y.F.); zhongcai.liao@heaye.com (Z.L.); min.wei@heaye.com (M.W.); 5 International Magnetobiology Frontier Research Center (iMFRC), Science Island, Hefei 230031, China

**Keywords:** static magnetic fields, diabetic wound healing, oxidative stress, NRF2, physical therapy

## Abstract

Although some studies have shown that some static magnetic fields (SMFs) can promote wound healing in diabetic mice, it is not clear whether the other diabetes complications, such as liver disease and diabetic nephropathy, can also be alleviated. Here, we constructed two simple magnetic plates using neodymium permanent magnets to examine the comprehensive effects of moderate SMFs on genetically obese leptin receptor-deficient db/db diabetic mice. We found that although the blood glucose was not obviously reduced by these two SMF settings, both of the glycated serum protein (GSP) and malondialdehyde (MDA) levels were significantly decreased (Cohen’s d = 2.57–3.04). Moreover, the wound healing, liver lipid accumulation, and renal defects were all significantly improved by SMF treatment (Cohen’s d = 0.91–2.05). Wound tissue examination showed obvious nuclear factor erythroid 2-related factor 2 (NRF2) level decrease (Cohen’s d = 2.49–5.40) and Ki-67 level increase (Cohen’s d = 2.30–3.40), indicating decreased oxidative stress and increased cell proliferation. In vitro cellular studies with fibroblast NIH3T3 cells showed that SMFs could reduce high glucose-induced NRF2 nucleus translocation (Cohen’s d = 0.87–1.15) and cellular reactive oxygen species (ROS) elevation (Cohen’s d = 0.92), indicating decreased oxidative stress. Consequently, high glucose-induced impairments in cell vitality, proliferation, and migration were all improved by SMF treatment. Therefore, our results demonstrate that these simple SMF devices could effectively reduce oxidative stress in diabetic mice and may provide a cost-effective physical therapy strategy to alleviate multiple diabetic complications in the future.

## 1. Introduction

According to the latest data of the International Diabetes Federation (IDF) in 2021, there are approximately 537 million adults living with diabetes mellitus (DM), a complex disease characterized by hyperglycemia (high blood glucose). Most diabetic patients have multiple skin, liver, and renal complications, which severely impaired their life quality. One of the most prevalent complications in diabetic patients is impaired diabetic wound healing [1]. In fact, the diabetic skin ulcers are usually very hard to heal, which cause infections, amputation, and even death [2,3,4].

Oxidative stress, a state when the balance between oxidative and antioxidant actions lean towards oxidation, and excessive reactive oxygen species (ROS), chemically reactive radicals or non-radical molecules derived from molecular oxygen [5], play important roles in the process of diabetic wound healing [6,7]. The hyperglycemic microenvironment of the wounds of diabetic patients is more prone to oxidative stress than that of the normal organism, since it is unfavorable for wound healing [8]. Excessive oxidative stress in the damaged wounds causes DNA damage [9], cellular senescence [10], fibroblast cell death [11,12], and inflammatory responses [13], which greatly hamper the wound healing process. The excessive ROS accumulation in the wound can inhibit the function of macrophages and angiogenesis, thus hindering wound tissue regeneration and blood vessel reconstruction [14,15]. Consequently, controlling ROS and oxidative stress level in the diabetic wound is an important strategy to promote wound healing. Guan et al. synthesized a material that could scavenge ROS from diabetic wounds, which promoted diabetic wound healing [16]. Zhao et al. prepared a ROS scavenging hydrogel to accelerate wound healing by reducing ROS levels [17]. Wu et al. synthesized a tissue adhesive nanocomposite, which can significantly suppress the deleterious effects of excess ROS produced at diabetic wound sites [18]. In addition, Chang et al. found that the matrix metalloproteinase 9 (MMP-9) inhibitor can accelerate wound healing in diabetic mice by reducing ROS and inflammation levels [19].

As a noninvasive physical treatment method, magnetic fields can control the movement and transfer of unpaired electrons in free radicals, which provide a theoretical physical basis for their regulation of ROS [20,21]. The effects of different static magnetic fields (SMFs) by ROS are changeable and mostly depend on SMFs parameters, including intensity and/or gradient [22,23]. Moreover, SMF direction has also been shown to have differential effects [24,25]. However, it is interesting that multiple studies show that the diabetic wound healing process could be promoted by different SMFs [26,27,28] and dynamic magnetic fields [29,30]. It has been proposed that 3 mT SMF combined with electric field can affect the generation of free radicals through radical-pair recombination [31]. A 0.389 T moderate SMF can also reduce oxidative stress and inflammatory responses induced by ragweed pollen extract in mice and human lung cancer cells [32]. These studies reveal that using SMFs to improve wound healing in diabetes patients seems to have a lot of potential in the future. Moreover, previous studies also show that 100 mT moderate SMF alone or 3 mT moderate SMF combined with electric field could reduce ROS level to reduce blood glucose and significantly alleviate type 2 diabetes (T2D) complications, including fatty liver and renal defects [31,33]. However, whether we can use a specific SMF device to improve diabetic wound healing as well as alleviate other diabetic complications simultaneously is still unknown. 

It has been shown that magnetic fields of hundreds of mTs may have some therapeutic effects, but the magnetic field direction, intensity, and treatment time could directly influence the results [22]. Therefore, we chose to use the permanent magnet cubes that have great potentials to be applied in the field of magnetotherapy in the future. In this work, we chose two differently oriented moderate SMFs to treat these mice to explore the influences of SMFs on blood glucose, serum biochemistry, wound healing, as well as liver and kidney condition in db/db diabetic mice. We also investigated the mechanisms by performing cellular assays using mouse embryo fibroblast cells. Our findings demonstrate that these moderate SMFs could improve multiple diabetic complications by reducing oxidative stress and improving cell vitality, which made SMFs a potential physical method to be used in diabetes treatment in the future.

## 2. Materials and Methods

### 2.1. Static Magnetic Field Exposure 

In mice experiments, db/db diabetic mice were housed in the cage placed on the top of the magnetic or nonmagnetic plate (Length × width: 310 × 250 mm). Our previous studies indicate that the magnetic pretreatment before the onset of diabetes is beneficial for the diabetic mice [33] so that we chose to pretreat these mice with SMFs for 7 weeks (24 h/day). After the pretreatment, the wounds of db/db mice were made and continuously exposed to SMFs for an additional 3 weeks (24 h/day). The magnetic plate consists of 8 cylindrical permanent magnets (diameter × height: 16 × 7 mm) embedded in a polyvinyl chloride board. The North pole and the South pole of the permanent magnets were arranged differently in the two plates, so that the mice were exposed to SMFs with two opposite directions (upward vs. downward). In this investigation, the North and South poles of the magnets were represented by the upward and downward directions, respectively. The magnetic field strengths at the mice’s locations reached ~ 15 mT in both directions. The sham groups were put in the identical nonmagnetic plate device to reduce experimental variability.

N38 Neodymium permanent magnets (length × width × height: 60 × 50 × 35 mm) offered 0.5 T SMF for cellular studies and the details of the magnetic apparatus have been detailed in prior work [33]. The control and sham groups were put in the same cell incubator, but far away from the magnets, to reduce experimental variability. The magnetic field strength of the control group is 0.0001 T, which is 5000 times less than the experimental group using a 0.5 T magnetic field. In addition, high-glucose-treated cells were continuously exposed to sham or SMFs for 12–48 h.

### 2.2. Animals

Thirty-three 4-week-old male BKS wild-type (WT) and BKS-Lepr^db^/J (db/db) mice were used in this study. Six healthy WT mice served as the control group. Twenty-seven db/db mice served as sham, upward SMF, and downward SMF group (N = 9 mice/group). All mice were purchased from GemPharmatech Co., Ltd. (GemPharmatech, Nanjing, Jiangsu, China). The mice were provided with standard rodent chow and water and housed in cages (3 mice per cage) that were placed in SPF animal facility with the laboratory maintained at a temperature of 22 °C and 40% humidity with a 12 h light: 12 h dark cycle. We recorded the changes in body weight and fasting blood glucose of mice during the whole experiment. All animal welfare and experimental procedures were performed strictly according to the National Institutes of Health Guide for the Care and Use of Laboratory Animals (NIH publications No. 8023, revised 1978). Moreover, the procedures were approved by the Institutional Animal Care and Use Committee of Hefei Institutes of Physical Science, Chinese Academy of Sciences (DWLL-2021-03, 22 January 2021).

### 2.3. Wound Healing Experiments

Cutaneous wounds were established as detailed by Shang et al. [26]. Briefly, mice were narcotized by intraperitoneal injection of 1% pentobarbital sodium; the dorsal surface was shaved, washed with povidone iodine solution, and cleaned with an alcohol swab. Two excisional wounds were made on each side of the midline of the shaved dorsum using a sterile 5 mm punch biopsy tool (Miltex, Princeton, NJ, USA). Wound sizes were assessed in the 4th, 9th, and 22nd day post-wounding by planimetry. Photographs of the wounds were taken with a transparent grid of known dimensions (length × width: 1 × 1 cm) placed on top of the wound. Wound areas were calculated by Image J software (National Institutes of Health, Bethesda, MD, USA), and wound closure was calculated as the percent area of the original wound. Here, to evaluate the impacts and mechanisms of SMFs on diabetic wound healing, we used male diabetic db/db mice, whereas wild-type mice were also chosen as a healthy group to investigate wound healing without diabetes and the treatment of a magnetic field. Five-week-old male db/db mice were treated for 7 weeks with ~15 mT upward or downward SMF, or un-magnetized magnetic plate, and then wound induction was performed. The wild-type male mice were given the same treatment as db/db mice, except they were kept away from the magnet.

### 2.4. Tissue Examinations

The mice were randomly sacrificed on day 4, 9, and 22 to collect samples including the injured skin and its surrounding tissues and organs, respectively. Samples were fixed in 4% paraformaldehyde. After being embedded with paraffin, the samples were sectioned with a microtome. The obtained sections (5 μm) were stained for Ki-67 (Proteintech, 1:2000, rabbit polyclonal antibody), hematoxylin and eosin (H&E), Sirius red, periodic acid-Schiff (PAS), Masson’s Trichrome-stained and/or oil red O. Slides were scanned using an Aperio Scanner (Aperio Technologies, Vista, CA, USA) and analyzed using CaseViewer software (3DHISTECH, Budapest, Hungary). Quantified analysis of images by Image J software (National Institutes of Health, Bethesda, MD, USA).

The malondialdehyde (MDA) level and superoxide dismutase (SOD) level of wound tissues were detected by SOD assay kit (Beyotime, Shanghai, China) and MDA assay kit (Beyotime, Shanghai, China). 

### 2.5. Serum Biochemistry

Three mice in each group were sacrificed on day 4, 9, and 22 to collect blood samples in 1.5 mL centrifuge tubes. To obtain serum, blood samples were centrifuged at 4000× *g* for 10 min and were analyzed using an automated biochemical analyzer (Rayto, Shenzhen, Guangdong, China).

### 2.6. Cell Culture and CCK-8 Cytotoxicity Assays

The mouse embryonic fibroblasts cells (NIH3T3) and fibroblast cells (L929) cells were from American Type Culture Collection (Manassas, VA, USA) and cultured in DMEM (Corning, Corning, NY, USA) or RPMI 1640 (Shanghai QiDa Biotechnology Co., Ltd., Shanghai, China) supplemented with 10% FBS, 2 mM GlutaMAX, and 1% penicillin/streptomycin. Control group cells were cultured with basal medium, and sham and SMF groups cells were cultured with medium supplemented with high glucose (50 mM). All cells were maintained at 37 °C under 5% CO_2_ in a humidified incubator (Thermo Fisher Scientific, Waltham, MA, USA). 

We used Cell Counting Kit-8 (CCK-8) (Boster, Wuhan, Hubei, China) to measure cell viability. The NIH3T3 cells were seeded in 96-well plates (5 × 10^4^ cells/mL), cultured in DMEM supplemented with increasing doses of glucose (25.02 mmol/L, 30 mmol/L, 50 mmol/L, 70 mmol/L) at 37 °C for 24 h before the addition of CCK-8 solution (10 μL/well). After another 1.5 h of incubation, we examined absorbance at 450 nm with a Microplate Reader (BioRad, Hercules, CA, USA).

### 2.7. Intracellular ROS Detection

The NIH3T3 cells in a 35 mm culture dish (4 × 10^5^ cells/mL) were cultured in DMEM including 50 mM glucose at 37 °C, whereas treated with sham, upward, or downward SMF for 24 h. Intracellular ROS can oxidize the DCFH-DA probe, resulting in fluorescent DCF that can be identified by flow cytometry (Beckman Coulter, Brea, CA, USA).

### 2.8. Immunofluorescence Staining

The NIH3T3 Cells and L929 cells were fixed with paraformaldehyde, treated with blocking buffer (0.2% sodium azide, 2% bovine serum albumin, and 0.1% Triton X-100 in TBS-Tween) for 1 h, and incubated with the primary antibodies at 4 °C overnight. The primary antibodies were diluted as follows: NRF2 (1:1000, Novoprotein, Suzhou, Jiangsu, China), Ki67 (1:1000, Proteintech, Chicago, IL, USA). Furthermore, the cellular nuclei were stained with DAPI (1:500, Sigma, Aldrich, MO, USA) for 8 min. Finally, slips were mounted in gold antifade reagent (Thermo Fisher Scientific, Waltham, MA, USA) and captured on a confocal microscope (Olympus, Tokyo, Japan).

### 2.9. Cell Scratch and Migration Assay

The NIH3T3 (4 × 10^5^ cells/mL) cells were planted in a 3.5 cm culture dish and cultured in total media until they reached confluency. The medium was replaced with a medium containing 0.1% serum and normal concentrations of glucose or 50 mmol/L glucose. Next, cell monolayers were scratched by 10 μL plastic tip to produce straight lines in the cell monolayers and exposed to sham, upward, or downward SMF, respectively. Finally, the distance between the scratches was photographed until recovery of the monolayer, and the results were analyzed by Image J software (National Institutes of Health, Bethesda, MD, USA).

The migration of NIH3T3 cells was assessed utilizing a Transwell tool (Corning, Corning, NY, USA). Almost 5 × 10^4^ cells were suspended in 100 μL serum-free medium and seeded onto the upper chambers. Then, 500 μL DMEM basic medium with 20% fetal bovine serum was added to the lower chambers. The prepared 24-well plates were exposed to sham, upward, or downward SMF, respectively. After incubation for 48 h at 37 °C under 5% CO_2_, the medium was removed from the upper chamber and the cells on the upper side of the chamber were scraped off with a cotton swab. The cells on the lower side of the upper chamber were fixed with 4% methanol solution and stained with 1% crystal purple solution. Take pictures by microscope (magnification 400×), then analyze cell numbers with Image J software (National Institutes of Health, Bethesda, MD, USA).

### 2.10. Nuclear and Cytoplasmic Protein Extraction

The nuclear and cytoplasmic protein extraction kit (Beyotime, Shanghai, China) was used to extract nuclear and cytoplasm proteins. Briefly, the NIH3T3 cells were collected into centrifuge tubes. The cells were homogenized and suspended in cytoplasmic protein extraction reagent and were centrifuged at 13,800× *g* for 5 min to extract cytoplasmic proteins. The pellet was resuspended in nuclear protein extraction reagent to lyse for 30 min and the supernatant was collected as nuclear protein after centrifugation at 13800× *g* for 10 min. Protein quantification was carried out using the BCA protein assay kit (Beyotime, Shanghai, China).

### 2.11. Western Blot Analysis

Equal quantities of the proteins were separated by SDS-PAGE gels, transferred to nitrocellulose membranes, incubated with 5% skimmed milk, and then incubated with the primary antibodies at 4 °C overnight. The primary antibodies were diluted as follows: NRF2 (1:1000, GeneTex, Irvine, CA, USA), β-tubulin (1:2000, ZenBio, Chengdu, Sichuan, China), and Lamin A/C (1:2000, Proteintech, Chicago, IL, USA). The membranes were incubated with the HRP-labeled secondary antibody in blocking buffer at room temperature. Blots were developed using an enhanced chemiluminescence reagent (Thermo Fisher Scientific, Waltham, MA, USA). The relative protein density was quantified using Image J software (National Institutes of Health, Bethesda, MD, USA).

### 2.12. Cell-Titer Glo Test

The NIH3T3 cells were incubated in 96-well plates under the previously described conditions for 24 h. According to the manuscript protocol, Cell-titer glo was applied to measure cell vitality (Promega, Madison, WI, USA).

### 2.13. Calcein Acetoxymethyl Ester and Propidium Iodide (Calcein-AM/PI) Staining

The Calcein-AM/PI stain kit (Beyotime, Shanghai, China) was used to distinguish between dead and live cells. The NIH3T3 cells were cultured in 96-well plates at 5000 per well and incubated with different concentrations of glucose (25.02 mM, 50 mM). Next, the cells were processed for 24 h in either the sham, upward, or downward SMF. Live cells stained by Calcein-AM emitted green fluorescence and dead cells stained by PI emitted red fluorescence.

### 2.14. EdU Flow Cytometry Test

The NIH3T3 cells were incubated in a 3.5 cm culture dish and incubated with different concentrations of glucose (normal, 50 mM). Next, the cells were processed for 24 h in either the sham, upward, or downward SMF. Then, the cells were incubated with EdU (5-ethynyl-2′-deoxyuridine, 10 µM) for 2 h. Subsequently, the cells were collected and measured by Click-iT EdU Flow Cytometry Assay Kits (Epizyme Biomedical Technology, Shanghai, China), as described by the manufacturer.

### 2.15. Statistical Analysis

Data from the experiments were shown as the mean ± SD and were analyzed by the two-tailed Student’s *t*-test. All the statistical analysis was made by GraphPad Prism 9 software (GraphPad Software, San Diego, CA, USA) and *p* value < 0.05 was considered as statistically significant.

## 3. Results

### 3.1. SMFs Alleviate Multiple Diabetic Complications

**Figure 1:**
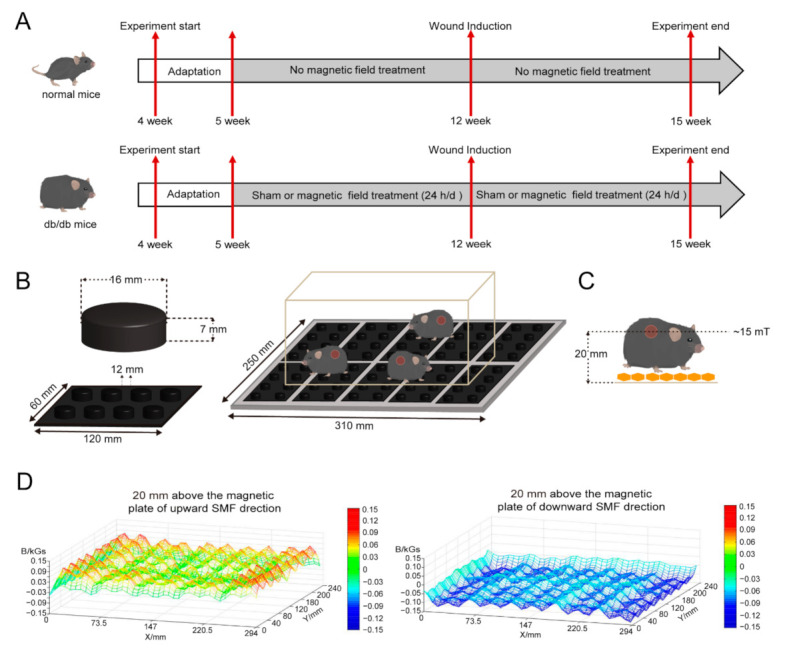
Experiments design and schematic view of the static magnetic field exposure system. (**A**) In vivo experiments design. (**B**) The device (length × width: 310 × 250 mm) consists of ten plates. Each plate (length × width: 120 × 60 mm) contains 8 cylindrical permanent magnets (diameter × height: 16 × 7 mm). Moreover, the magnets were placed next to each other with the same orientation. The cylindrical permanent magnets were mounted in a polyvinyl chloride board with a 12 mm separation between them. The whole cage was placed on the magnetic plate. (**C**) The magnetic field strength is ~15 mT at 20 mm above the magnetic plate, where the mice wound located. (**D**) Devices with different directions of magnetic plates and magnetic field intensities at the positions of the mice in each exposure condition are provided and measured.

To investigate the effects of SMFs on diabetic wound healing and other diabetic complications, a sham and two different magnetic plates were used (Figure 1A). We used magnetic field devices, and the magnetic field treatment modalities are shown in Figure 1B. Then, we used an automated magnet analyzer to measure the magnetic field distributions at the horizontal levels where the mice wounds were located (approximately 20 mm above the magnetic plate, Figure 1C). The average peak intensities of the upward and downward SMF at the wounds were ~15 mT (Figure 1D).

**Figure 2:**
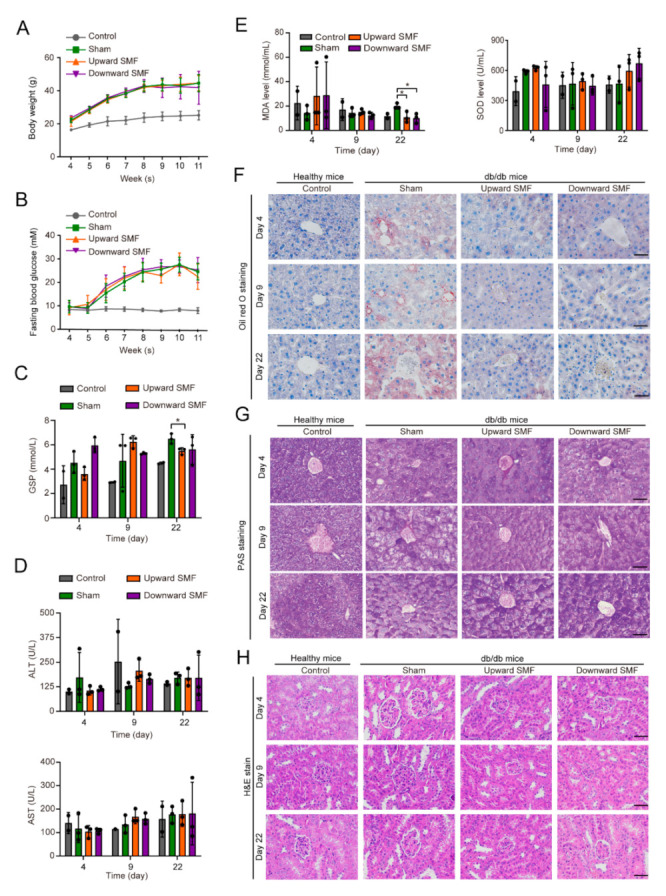
SMFs alleviate multiple diabetic complications. (**A**) Body weight and (**B**) fasting blood glucose level measurement in the control group and db/db mice groups treated with sham, upward or downward SMF (Control, N = 6 mice; Sham, N = 9 mice; Upward SMF, N = 9 mice; Downward SMF, N = 9 mice). (**C**) Glycated serum protein (GSP), (**D**) alanine aminotransferase (ALT) and aspartate aminotransferase (AST), (**E**) malondialdehyde (MDA) and superoxide dismutase (SOD) levels were measured on day 4, 9, and 22 post-wounding (Control, N = 2 mice; Sham, N = 3 mice; Upward SMF, N = 3 mice; Downward SMF, N = 3 mice). (**F**) Oil red O and (**G**) PAS staining of liver tissues of control or db/db mice. Scale bar = 50 µm. (**H**) H&E stains of kidney sections on the 4th, 9th, and 22nd day. Scale bar = 50 µm. Values were expressed as mean ± SD. * *p* < 0.05.

We monitored the mice body weight (Figure 2A) and fasting blood glucose levels (Figure 2B) throughout the experiments and performed blood biochemistry analysis on the 4th, 9th, and 22nd day post-wounding (Figure 2C–E, Supplementary Materials Figure S1). Although no significant differences were found in their body weight or fasting blood glucose levels, the glycated serum protein (GSP) level was decreased by the end of the experiment (Cohen’s d = 2.91) (Figure 2C). Most blood biochemistry indicators were not significantly changed by SMFs (Figure 2D, Supplementary Materials Figure S1), except the malondialdehyde (MDA) (Cohen’s d = 2.57–3.04), creatinine (Cohen’s d = 2.61), and low density lipoprotein cholesterol (LDL-c) (Cohen’s d = 3.79) (Figure 2E, Supplementary Materials Figure S1). At the end of the experiment, the MDA levels were decreased by approximately 45% (*p* < 0.05) and the superoxide dismutase (SOD) levels were also slightly increased (*p* > 0.05), which indicate the lipid peroxidation and the oxidative stress level in these db/db mice were alleviated by SMFs treatment.

To evaluate the effect of SMFs on other complications, we extracted the liver and kidney from mice for tissue analysis. The hematoxylin and eosin (H&E) staining for liver showed that the hepatocytes of db/db mice in the sham group were loosely arranged and had a large number of vacuoles, which were obviously alleviated by SMF treatment (Supplementary Materials Figure S2). We used oil red O staining to stain fat deposition and found that these SMFs significantly reduced the lipid accumulation in liver (Figure 2F). We also performed periodic acid-Schiff (PAS) staining to examine the capacity of glycogen synthesis in the liver and found that SMFs significantly rescued the diabetes-induced PAS staining decreasing on days 9 and 22 (Figure 2G). In addition, The H&E staining for kidney showed that the diabetic mice had multiple kidney abnormities, including glomerular augmentation, mesangial matrix thickening, and dilated tubular lumen with vacuolization, which were alleviated by SMFs treatment (Figure 2H).

### 3.2. SMFs Decrease NRF2 and Increase Ki-67 to Accelerate Wound Healing in Diabetic Mice

**Figure 3:**
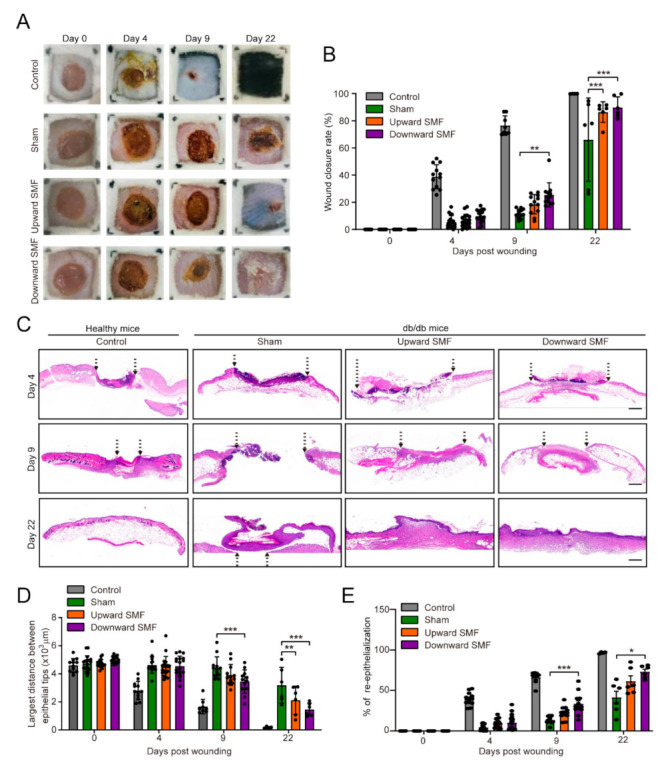
SMFs accelerate the healing effects on a chronic diabetic wound model. (**A**) Representative images of circular shaped full-thickness wound repair taken on day 0, 4, 9, and 22 post-wounding (a transparent grid (1 cm × 1 cm) was placed on the wound to quantify the area of the wound). (**B**) Quantification of wound closure rate. N = 6–9 mice, *n* = 12–18 wounds (0-day); N = 6–9 mice, *n* = 12–18 wounds (4-day); N = 4–6 mice, *n* = 8–12 wounds (9-day); N = 2–3 mice, *n* = 4–6 wounds (22-day). (**C**) Representative pictures of sections stained with H&E from wounded skin of WT mice group and T2D mice group treated as indicated on day 4, 9, and 22 post-wounding. Scale bar = 500 µm. Graphs show the distance between the epithelial tips of punched wound (**D**) and the percentage of wound re-epithelialization (**E**) in the healthy and diabetic groups recorded at different time points. N = 6–9 mice, *n* = 12–18 wounds (0-day); N = 6–9 mice, *n* = 12–18 wounds (4-day); N = 4–6 mice, *n* = 8–12 wounds (9-day); N = 2–3 mice, *n* = 4–6 wounds (22-day). Values were expressed as mean ± SD. * *p* < 0.05, ** *p* < 0.01 and, *** *p* < 0.001.

We examined wound status on day 4, 9, and 22 after wounding and found that SMF treatment significantly increased the wound area closure rate in these diabetic mice (Cohen’s d = 0.91–2.05) (Figure 3A,B). Since granulation tissue formation and re-epithelialization are usually used to reveal the status of cell proliferation and migration at the wound site [34], we used these indicators to evaluate the wound healing rate in H&E tissue examination. Our results showed that the db/db mice in the SMF group formed granulation tissue and re-epithelialization more obvious on 9- and 22-day than in the sham control (Cohen’s d = 1.23–2.18) (Figure 3C–E). Moreover, it is interesting that although both upward and downward SMFs promoted the wound healing process, the downward SMF had a more obvious effect (Cohen’s d = 1.23–2.18) (Figure 3D,E).

**Figure 4:**
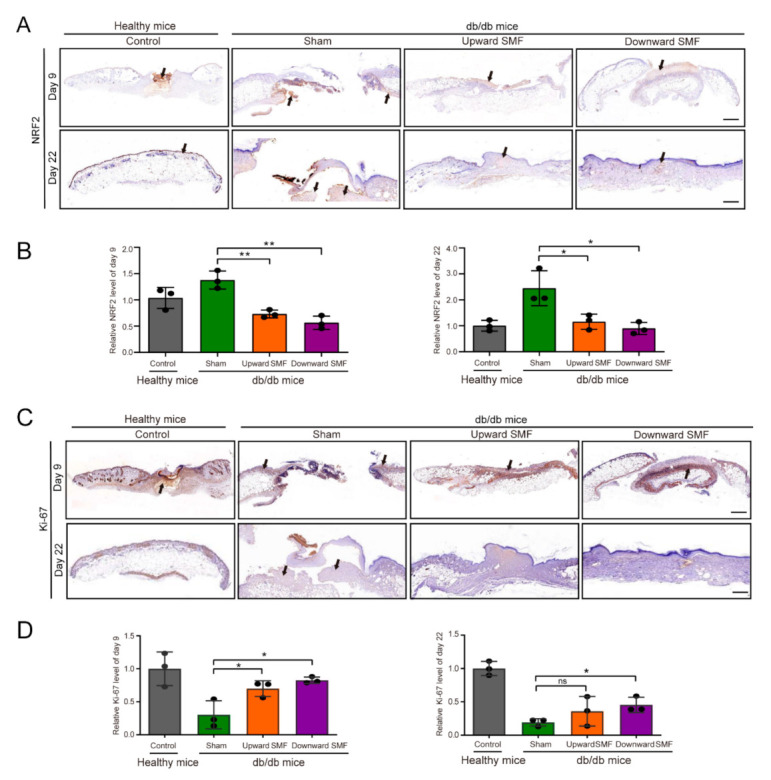
SMFs reduce NRF2 and increase Ki-67 levels in wound tissues. (**A**) Photomicrographs of NRF2 stained sections on the 9th and 22nd day. Scale bar = 500 µm. (**B**) Quantification of NRF2 levels at the wound tissue (N = 3 mice/group). (**C**) Photomicrographs of Ki-67-stained sections on the 9th and 22nd day. Scale bar = 500 µm. (**D**) Quantification of Ki-67 levels at the wound tissue (N = 3 mice/group). Values were expressed as mean ± SD. ns: not significant; * *p* < 0.05, and ** *p* < 0.01.

To study the mechanism by which SMFs promoted wound healing, we evaluated NRF2 (Figure 4A,B) and Ki-67 (Figure 4C,D) as cellular oxidative stress and proliferation markers at the wound site of db/db mice. Our immunohistochemical analysis results showed that the NRF2 level was significantly decreased on days 9 and 22 post wounding (Cohen’s d = 4.90–5.40) (Figure 4B) and the Ki-67 level in SMF-treated mice was much higher than mice in the sham group (Cohen’s d = 2.30–3.40) (Figure 4D). Similarly, Sirius red staining experiment results showed that numerous necrotic areas (yellow) and fewer collagen fibrils (red) in the sham group, whereas the SMFs treated group had fewer necrotic areas and numerous collagen fibers (Supplementary Materials Figure S3A). Consistent with the results of the Sirius red experiment, results of Masson’s Trichrome staining suggested that there were fewer necrotic areas and more collagen fibers at the wound tissues of the SMFs groups compared with the sham group, and epidermal layer tissues of the upward SMF group recovered faster (Supplementary Materials Figure S3A). Moreover, SMFs also increased SOD and decreased MDA levels in the wound tissues of diabetic mice (Supplementary Materials Figure S3B,C), but there was no statistically significance (*p* > 0.05) except for the downward SMF to SOD level (*p* < 0.05) (Cohen’s d = 1.10). This indicated that the oxidative stress and the lipid peroxidation in diabetic mice at the wound tissue were reversed by SMF treatment. Therefore, our animal experiments showed that SMFs could reduce oxidative stress and promote cell proliferation in the wounds of diabetic mice.

### 3.3. SMFs Reduce High Glucose-Induced Cellular Oxidative Stress and Improve Cell Vitality, Proliferation, and Migration

**Figure 5:**
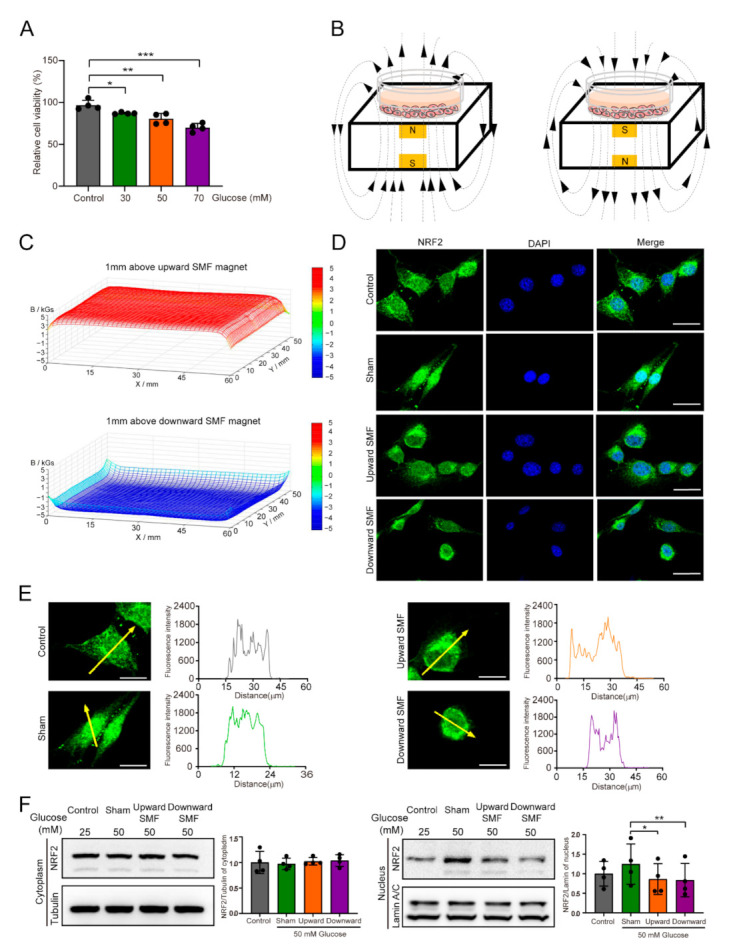
SMFs reduce high glucose induces NRF2 nuclear translocation. (**A**) Cell viability assay. (**B**) Graphs show examples of upward and downward magnetic fields. (**C**) The device of different directions magnetic block and magnetic field strengths at cell sites were measured. (**D**) Immunofluorescence staining of NRF2 was performed to show the basic expression levels and the locations of the proteins in the NIH3T3 cells. NRF2 staining is shown in green, and nuclear DNA staining by DAPI is shown in blue. Scale bar = 20 µm. (**E**) Distribution locations and quantifications of NRF2. Scale bar = 20 µm. (**F**) Expression levels of NRF2 of cytoplasm and nuclear levels were measured by Western blotting and quantified in NIH3T3 cells. Values were expressed as mean ± SD. * *p* < 0.05, ** *p* < 0.01, and *** *p* < 0.001.

Next, we used NIH3T3 cells to explore the effects of SMFs on high-glucose-treated cells in vitro. It is obvious that high glucose could decrease cell viability in a concentration dependent manner (Figure 5A). Using two permanent magnets (Figure 5B) that provided moderate SMFs with two different directions (Figure 5C), we found that SMF could reverse high glucose induced NRF2 nuclear import (Figure 5D,E), an indicator for increased intracellular oxidative stress in response to high glucose stimulated environments [35,36]. In order to verify this phenomenon, we also used L929 cells and observed similar results (Supplementary Materials Figure S4). Moreover, we further confirmed that the level of NRF2 in the nucleus could be significantly reduced by SMF treatment by Western blotting (Cohen’s d = 0.87–1.15) (Figure 5F).

**Figure 6:**
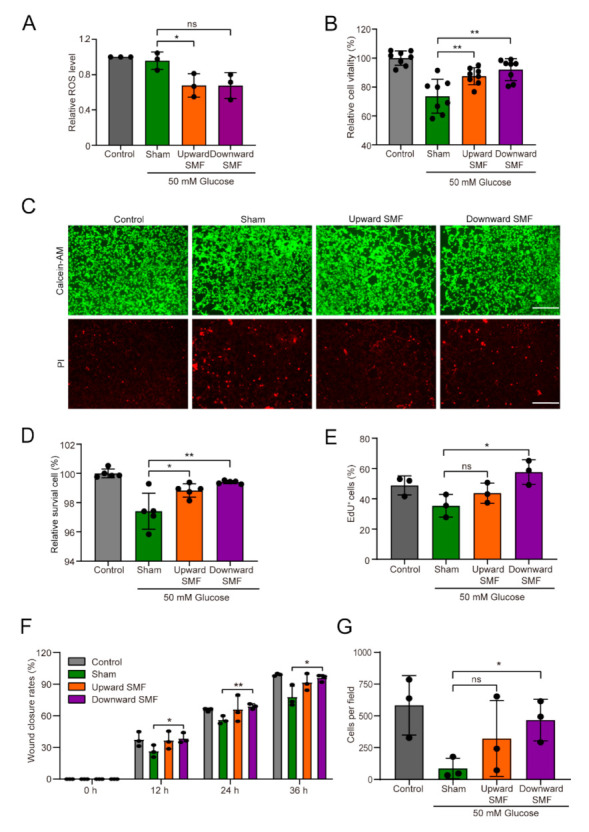
SMFs decrease cellular oxidative stress levels and improve cell vitality, wound closure rate, and cell migration. (**A**) ROS assay by Flow cytometry. (**B**) The results were detected by Cell-Titer Glo test. (**C**,**D**) Representative fluorescence images and quantification of Calcein-AM/PI staining. Scale bar = 300 µm. (**E**) Proliferation capacity of NIH3T3 cells assessed by Click-iT EdU Flow Cytometry Assay. (**F**) Quantification of scratch wound closure assay. (**G**) Quantification of Transwell cell migration assay. Values were expressed as mean ± SD. ns: not significant; * *p* < 0.05, and ** *p* < 0.01.

Since NRF2 is a crucial controller of the cytoprotective reaction, which is closely related to cellular ROS or oxidative stress [37,38], we next investigated the effect of SMFs on ROS levels. Obviously, SMFs could decrease the cellular ROS levels (Cohen’s d = 0.92) (Figure 6A), and could improve cell vitality (Cohen’s d = 0.60–2.27) (Figure 6B–D) and cell proliferation (Cohen’s d = 2.86–5.28) (Figure 6E, Supplementary Materials Figure S5) of NIH3T3 cells. In addition, we performed scratch wound closure assay (Figure 6F, Supplementary Materials Figure S6A) and Transwell assay (Figure 6G, Supplementary Materials Figure S6B) to investigate the influence of SMFs on the migration of NIH3T3 cells. As expected, we found that SMFs significantly promoted cell migration, especially after downward SMF treatment (Cohen’s d = 2.27–4.11) (Figure 6F,G).

## 4. Discussion

**Figure 7:**
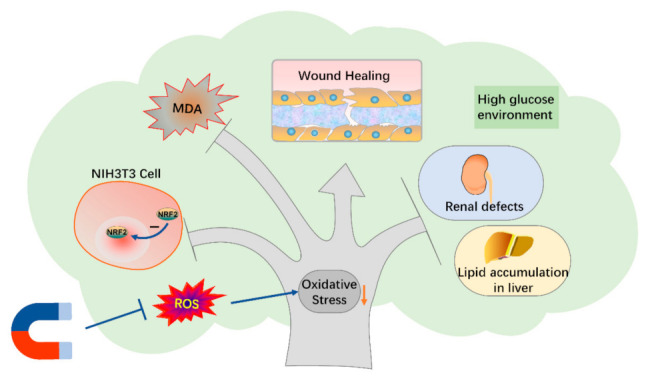
The mechanism of diabetic complications with SMFs treatment.

SMFs have been shown to have differential effects on blood glucose levels. For example, recently, Carter et al. used a combined SMF and electric field [31] and our group used a downward SMF of ~0.1 T to efficiently decrease blood glucose levels in T2D mice [33]. In the meantime, there are also some studies showing opposite or no effects of SMFs on the blood glucose [28,39,40,41]. The beneficial impacts of SMFs on wound healing, on the other hand, are consistent in all reported studies [30]. Moreover, there are also a few studies reported that some SMFs could improve diabetic neuropathy [42] and diabetic osteoarthropathy [43]. The purpose of our research was to study the comprehensively influences of some simple magnetic field devices on T2D mice. Our in vitro results showed that moderate SMFs reduced oxidative stress to increase cell vitality, promote cell proliferation and migration. Consequently, moderate SMFs might help db/db mice with fatty liver and renal defects, as well as promote wound healing (Figure 7). The lack of effective improvement of these SMF settings on blood glucose is possibly due to the db/db mice we used, which is genetically obese leptin-receptor-deficient and have much more severe symptoms than high-fat-diet-induced diabetic mice models.

Although ROS and RNS (reactive nitrogen species) are essential for maintaining redox homeostasis in living organisms, excessive ROS and RNS can cause cytotoxicity and oxidative stress, a negative effect produced by free radicals in the body and is regarded to be an important factor in aging and various diseases. ROS plays a crucial part in wound healing, whereas excessive ROS level could induce DNA damage, protein structure changes, lipid superoxidase (MDA), and reduced glutathione-oxidized glutathione (GSH-GSSG) alterations [44]. Moreover, ROS-induced oxidative stress is also a major factor that hampers wound healing [15]. Our results demonstrated that moderate SMFs could effectively reduce high glucose-induced NRF2 protein translocation into the nucleus in both NIH3T3 and L929 fibroblast cells, which indicated that the high glucose-induced oxidative stress was reversed by SMFs. NRF2 is a critical factor of the cytoprotective reaction, which is crucial for reinstating cellular redox homeostasis [37]. As a master regulator of the antioxidant response, the increase in ROS cause NRF2, initially localized in the cytoplasm, to enter the nucleus and activate the transcription of ROS-detoxifying enzymes and antioxidant genes, in order to participate in the regulation of ROS levels [45,46,47,48]. For example, NRF2 can affect detoxification enzymes to reduce intracellular ROS caused by alcohol-induced oxidative stress [49]. Furthermore, NRF2 is a multifunctional factor that serves as both oxidative stress indicator as well as anti-oxidant. On the one hand, under oxidative stress, NRF2 will serve as an anti-oxidant and increase SOD transcription, on the other hand, after SOD increases and reduces the oxidative stress, the NRF2 will decrease consequently, reflecting lower oxidative stress [50]. Our results showed that SOD increased and NRF2 decreased in cells and mice after SMF exposure, indicating that the organism was under a low level of oxidative stress.

It has been shown that SMFs could affect cellular ROS levels. On one hand, it may be related to different magnetic field parameters (intensity, direction, gradient, and exposure time, etc.). For example, exposure to 1 T SMF for one day decreased ROS levels [51], whereas exposure to 10 mT SMF for one day increased ROS levels in MCF-7 cells [52]. 60 mT SMF treatment for 45 min resulted in elevated ROS levels, whereas treatment for 15 min significantly lowered ROS levels in peripheral blood neutrophils [53]. On the other hand, there are many differences in various biological samples. Exposure to 1 T SMF for 1 day significantly reduced cellular ROS levels in HSAEC-30KT and HepG2 cells; however, the same magnetic field treatment conditions did not alter ROS of NIH3T3 cells [51]. We speculate that magnetic fields may alter the electron spin states of metabolic intermediates in the organism, thus affecting chemical reactions and leading to different changes in ROS levels.

In this research, we found that the downward SMF was more effective in promoting the wound healing and re-epithelialization than the upward SMF. In addition, in vitro experiments were consistent with the in vivo results that the downward SMF was more effective in promoting the proliferation, migration, and survival of cells than upward SMF. As one of the parameters of the magnetic field, although the mechanism is still poorly explored, magnetic field direction has been shown by multiple studies to influence biological effects [25,33,54,55]. Our previous study showed that the upward, but not downward, high intensity SMFs can affect DNA movement and supercoil tightness via Lorentz force acting on the negatively charged DNA, which consequently inhibited DNA synthesis and tumor cell proliferation [54,55]. More mechanistic studies are needed to unravel the various differential effects caused by upward vs. downward SMF direction.

## 5. Conclusions

In conclusion, our study revealed that SMFs could reduce ROS and oxidative stress, which further decreased the nuclear NRF2 translocation and the MDA level, promoted diabetic mice wound healing, and reduced liver lipid accumulation and renal defects. Our study demonstrates that SMFs provided by permanent magnets is promising as a low-cost physical tool to ameliorate diabetes induced chronic skin damage, liver, and kidney injury, as well as other oxidative stress-related health conditions in the future.

## Data Availability

The data used to support the findings of this study are included within the article.

## Supplementary Materials

The following supporting information can be downloaded at: https://www.mdpi.com/article/10.3390/cells11030443/s1, Figure S1: Effect of SMFs exposure on blood biochemistry test indicators in db/db mice, Figure S2: SMFs alleviate liver and kidney damages in db/db mice, Figure S3: SMFs accelerate different aspects of wound healing in db/db mice, Figure S4: Expression and distribution of NRF2 in L929 cell line, Figure S5: The SMFs promote the proliferation of high-glucose-treated NIH3T3 cells, Figure S6: SMFs promote wound closure and migration of NIH3T3 cells.
