# Supplementary material for: Static Magnetic Fields Reduce Oxidative Stress to Improve Wound Healing and Alleviate Diabetic Complications
Source: Cells. 2022 Jan 27;11(3):443. doi: 10.3390/cells11030443 (PMC8834397; doi:10.3390/cells11030443)
Supplement: Supplementary file 1 [file cells-11-00443-s001.zip › cells-1523802-supplementary.pdf]

cells

MDPI

Supplementary Material

Static Magnetic Fields Reduce Oxidative Stress to Improve Wound Healing and Alleviate Diabetic Complications

Chuan-Lin Feng[1,2,6], Biao Yu[2,3,6], Chao Song[2,3], Jun-Jun Wang[2], Lei Zhang[2], Xin-Miao Ji[2], Ying Wang[2,3], Yan-Wen Fang[4], Zhong-Cai Liao[4], Min Wei[4], and Xin Zhang[1,2,3,5*]

[1] Institutes of Physical Science and Information Technology, Anhui University, Hefei, 230039, P. R. China.

[2] High Magnetic Field Laboratory, Hefei Institutes of Physical Science, Chinese Academy of Sciences, Hefei, 230031, P.R. China.

[3] Science Island Branch of Graduate School, University of Science and Technology of China, Hefei, 230026, P. R. China.

[4] Heye Health Technology Co. Ltd, Huzhou, 313300, P.R.China.

[5] International Magnetobiology Frontier Research Center (iMFRC), Science Island, Hefei, 230031, P. R. China

[6] These authors contributed equally.

*Correspondence to: Xin Zhang, Ph.D. E-mail: xinzhang@hmfl.ac.cn

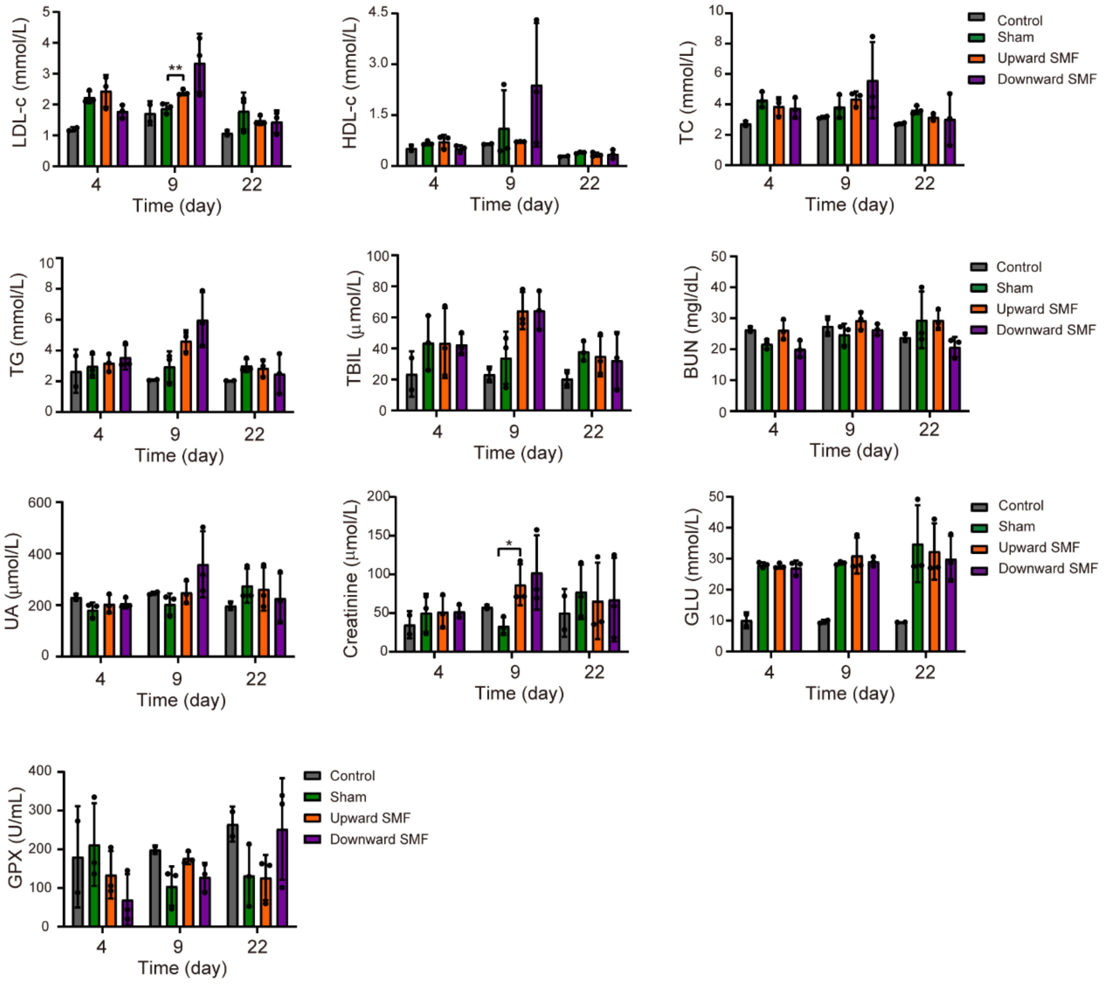

Figure S1. Effect of SMFs exposure on blood biochemistry test indicators in db/db mice. Serum low density lipoprotein cholesterol (LDL-c), high density liptein cholesterol (HDL-c), total cholesterol (TC), triglyceride (TG), total bilirubin (TBIL), blood urea nitrogen (BUN), uric acid (UA), creatinine, blood glucose (GLU) and glutathione peroxidase (GPX) were measured. Values were expressed as mean ± SD (Control, N = 2 mice; Sham, N = 3 mice; Upward SMF, N = 3 mice; Downward SMF, N = 3 mice). $^{*}p < 0.05$, *and* $^{**}p < 0.01$.

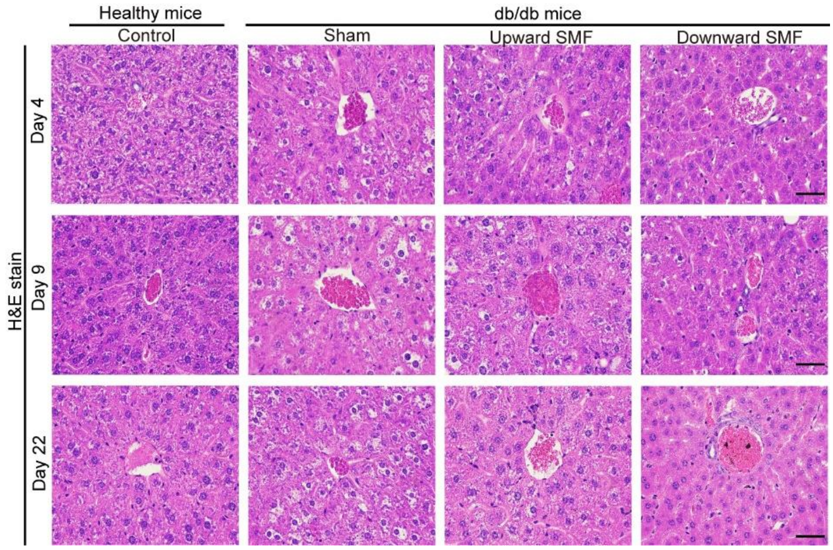

Figure S2. SMFs alleviate liver damages in db/db mice. Hematoxylin-eosin (HE) stains of liver sections on the 4th, 9th, and 22nd day. Scale bar = 50 μm.

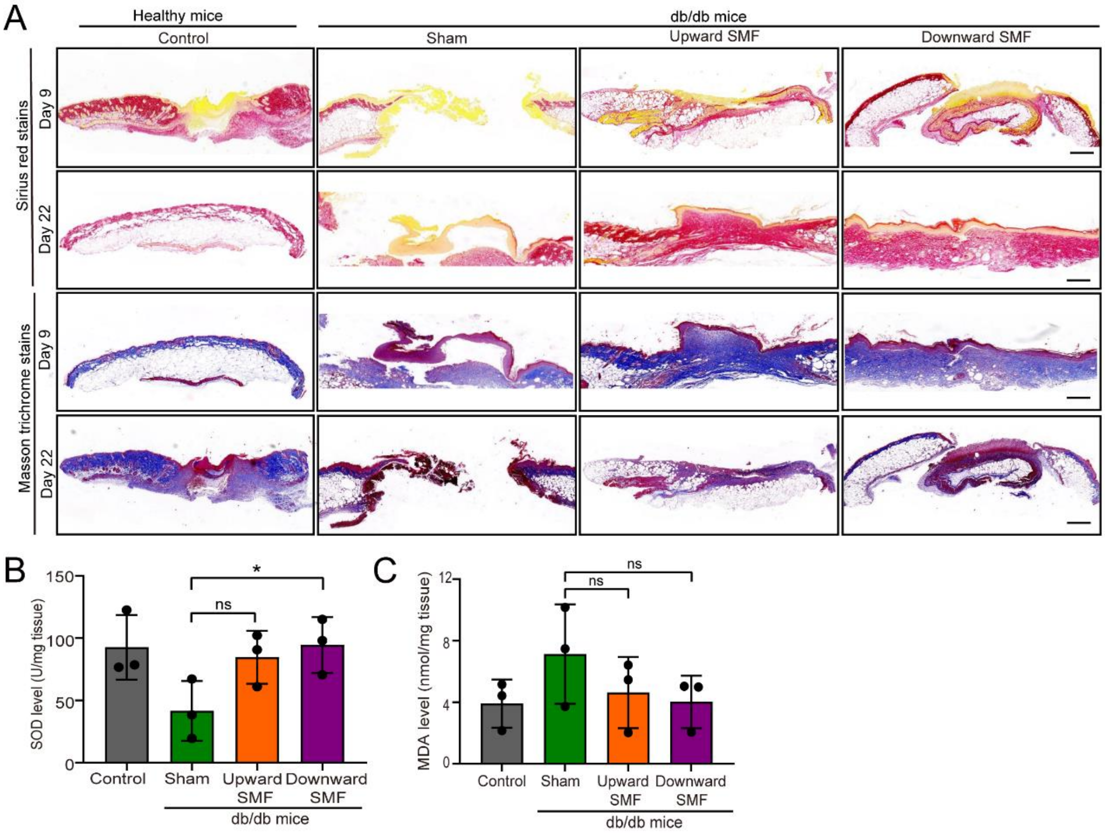

Figure S3. SMFs accelerate different aspects of wound healing in db/db mice. (A) Photomicrographs of Sirius red- and Masson's Trichrome-stained sections on 9th day and 22nd day. Scale bar = 500 μm. (B, C) Total superoxide dismutase (SOD) and malondialdehyde (MDA) in wound tissues were detected by the total Superoxide dismutase assay kit and lipid peroxidation malondialdehyde assay kit (N = 3 mice/group). Values were expressed as mean ± SD. *ns*: not significant; **p* < 0.05.

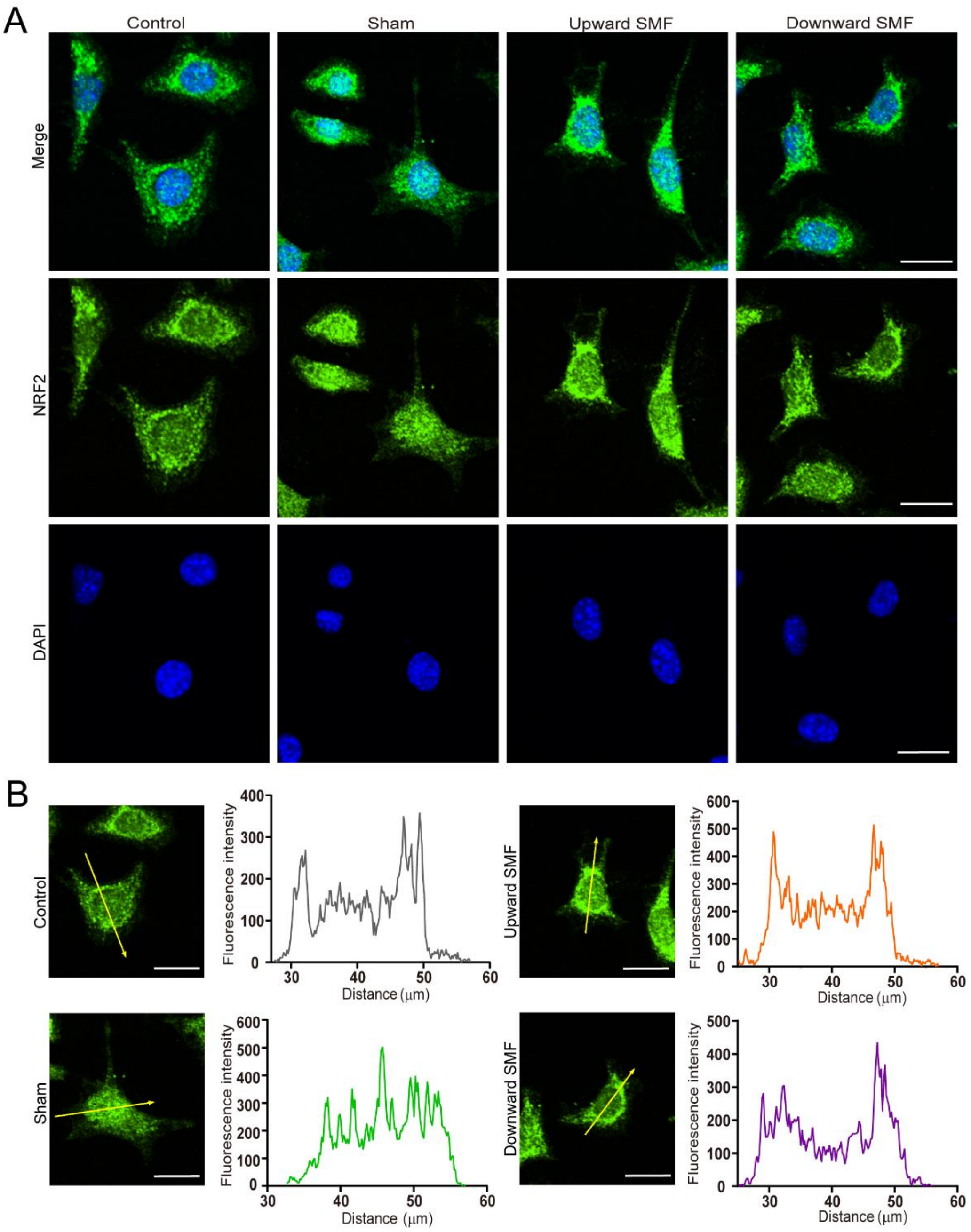

Figure S4. Expression and distribution of NRF2 in L929 cell line. (A) Immunofluorescence staining of NRF2 was performed to show the expression levels. Scale bar = 20 μm. (B) Distribution locations and quantifications of NRF2 in the L929 cells. NRF2 staining is shown in green, and nuclear DNA staining by DAPI is shown in blue. Scale bar = 20 μm.

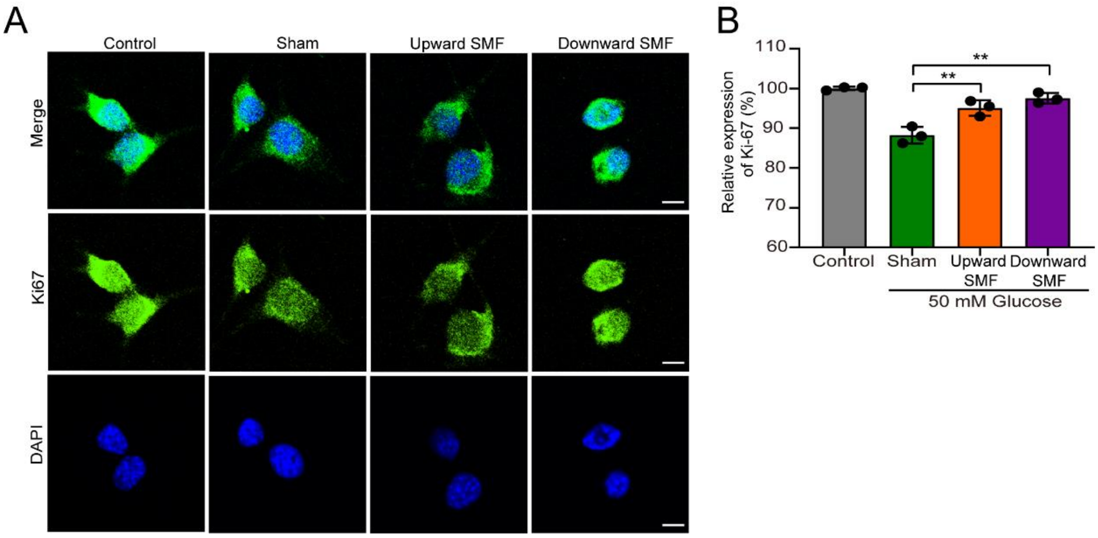

Figure S5. The SMFs promote the proliferation of high-glucose treated NIH3T3 cells. (A) Representative images of immunofluorescence staining of Ki-67. Scale bar = 20 μm. (B) Quantification of the percentage of Ki-67 fluorescence intensity in NIH3T3 cells. Values were expressed as mean ± SD. ***p* < 0.01.

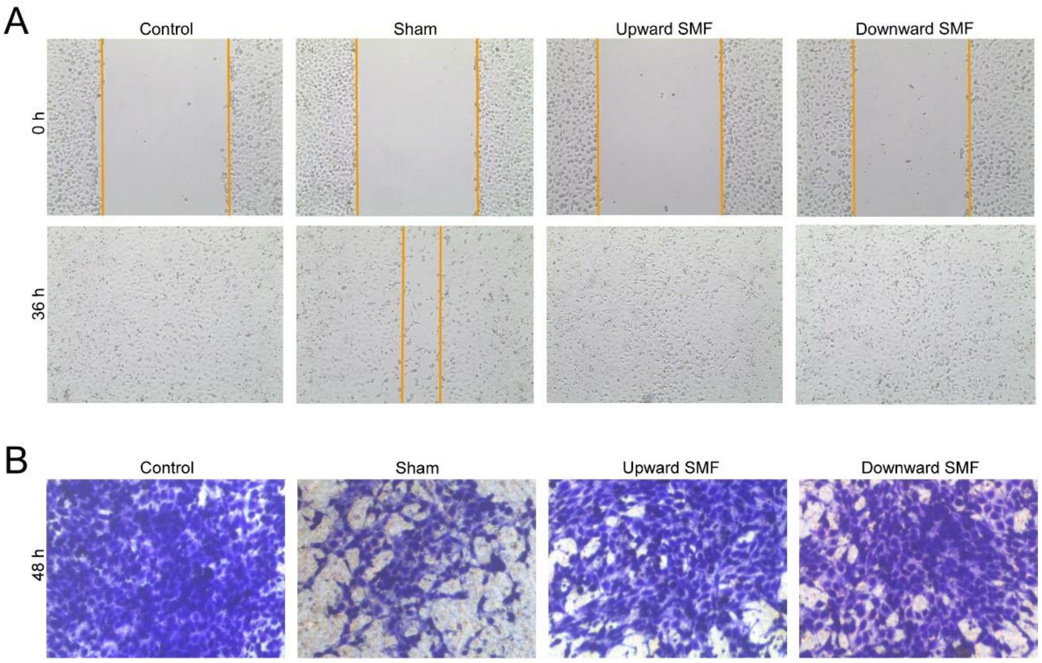

Figure S6. SMFs promote wound closure and migration of NIH3T3 cells. (A)Representative images of in vitro scratch wound closure assay. Magnification × 200. (B) Transwell assay analyses of the migratory capacity of NIH3T3 cells. Magnification × 400.
